# Oridonin suppresses autophagy and survival in rheumatoid arthritis fibroblast-like synoviocytes

**DOI:** 10.1080/13880209.2020.1711783

**Published:** 2020-01-23

**Authors:** Shou-Di He, Sheng-Guang Huang, Hui-Jun Zhu, Xiao-Guang Luo, Kang-Han Liao, Jie-Yao Zhang, Ning Tan, De-Yu Li

**Affiliations:** Traditional Chinese Medicine Department of Rheumatism, Huazhong University of Science and Techology Union Shenzhen Hospital, Shenzhen Nanshan People's Hospital, Shenzhen, China

**Keywords:** Chloroquine Oridonin, rheumatoid arthritis, autophagy, chloroquine

## Abstract

**Context:**

Oridonin exhibits various pharmacological and physiological activities, including antioxidant, antibacterial, anti-inflammatory, pro-apoptotic, anticancer and neurological effects. However, its role in rheumatoid arthritis (RA) is yet to be revealed.

**Objective:**

We evaluated the effects of oridonin on the survival and autophagy of RA-fibroblast-like synoviocytes (FLSs).

**Materials and methods:**

RA-FLSs were treated with oridonin at serial concentrations of 0, 2, 4, 6, 8 and 10 µg/mL for 24, 48 and 72 h. Then, cell proliferation and apoptosis were measured. A GFP-LC3 plasmid was transfected into the cells to determine autophagy.

**Results:**

Oridonin suppressed RA-FLS proliferation in a dose-dependent manner. The half maximal inhibitory concentrations (IC_50_) of oridonin at 24, 48 and 72 h were 8.28, 7.88 and 8.35 µg/mL, respectively. Treatment with oridonin for 24 h increased apoptosis by 4.1%, and increased the protein levels of Bax and cleaved caspase-3 but significantly decreased the levels of IL-1β in the culture supernatant (*p* < 0.05). In addition, 6 h of oridonin treatment significantly decreased the number of GFP-LC3 punctate dots and inhibited the protein levels of ATG5 and Beclin1 by 80.01% and 42.12%, respectively. Chloroquine (CQ) significantly reinforced the effects of oridonin on inhibition of autophagy, suppression of proliferation, and induction of apoptosis in RA-FLSs (*p* < 0.05).

**Conclusions:**

Our results indicate that treatment with oridonin in combination with CQ inhibits autophagy and cell proliferation and induces apoptosis in RA-FLSs more effectively than treatment oridonin alone. This finding indicates that oridonin is a potential therapeutic agent for RA.

## Introduction

Rheumatoid arthritis (RA) is a systemic autoimmune disease that causes the release of multiple inflammatory cytokines and matrix-degrading enzymes, ultimately leading to irreversible joint destruction and loss-of-function (Lefevre et al. 2009). Cartilage degradation, synovial hyperplasia and cellular infiltration of multiple cell types are the pathological hallmarks of RA (Humby et al. 2007). RA affects nearly 0.5–1% of the world’s population (Gibofsky 2012). In China, approximately five million people are newly diagnosed with RA each year (Yu et al. 2018). Presently, RA treatment relies on nonsteroidal anti-inflammatory drugs, steroids, disease-modifying anti-rheumatic drugs and biologic response modifiers, which often exhibit potential biotoxicity (Tlustochowicz et al. 2018).

Fibroblast-like synoviocytes (FLSs) are cells derived from the mesenchyme that line the internal synovium and play an important role in RA progression (Bartok and Firestein 2010). FLSs directly participate in synovial hyperplasia (Tu et al. 2018). In the synovial tissues of patients with RA, RA-FLSs proliferate aberrantly when stimulated with inflammatory cytokines such as IL-1, IL-6 and TNF-α (McInnes et al. 2000; Okamoto et al. 2008; Bartok and Firestein 2010). Additionally, RA-FLSs exhibit resistance to apoptosis (Alsaleh et al. 2016; Jiao et al. 2018; Kim et al. 2018). This dysregulation of RA-FLSs contributes to hyperplasia of the synovial lining and the infiltration of inflammatory cells into the sub-lining. Furthermore, RA-FLSs release multiple factors, including inflammatory cytokines, chemokines and matrix-degrading enzymes such as TNF-α, IL-1β, prostanoids and matrix metallo-proteinases, leading to the modulation of immune cells and proteolytic destruction of the extracellular matrix, cartilage and bone (McInnes et al. 2000; Okamoto et al. 2008; Bartok and Firestein 2010). Recently, RA-FLSs were recognized as novel therapeutic targets (Mor et al. 2005; Sacre et al. 2005).

Autophagy is an evolutionarily conserved secondary lysosomal degradation process by which cells degrade and recycle intracellular components in response to environmental stress (Mizushima 2007). Autophagy is critical for cellular homeostasis, innate and adaptive immune responses, and the regulation of inflammation (Deretic and Levine 2009; Dokladny et al. 2013). Recent studies have shown that dysregulation of autophagy occurs in rheumatic diseases (Dokladny et al. 2013; Rockel and Kapoor 2017; Hua et al. 2018), including systemic lupus erythematosus, osteoarthritis and RA. In patients with RA, autophagy is dramatically enhanced in the synovial linings of the synovial tissues and in RA-FLSs (Shin et al. 2010; Yang et al. 2017; Zhu et al. 2017). In addition, the inhibition of proteasomal activity in RA-FLSs increases LC3-II protein levels and prolongs RA-FLS cell survival (Kato et al. 2014), indicating that increased autophagy in RA-FLSs promotes RA-associated synovitis.

Oridonin, a bioactive diterpenoid isolated from *Rabdosia rubescens* (Benth.) Miq. (Myrtaceae), exhibits various pharmacological and physiological activities, including antibacterial, anti-inflammatory, pro-apoptotic, anticancer and neurological effects (Ikezoe et al. 2003; Li et al. 2011; He et al. 2018). Oridonin stimulates apoptosis in lung cancer, colorectal cancer, hepatocellular carcinoma and RA-FLSs (Ikezoe et al. 2003; Li et al. 2011). In the present study, we explored the effects of oridonin on the proliferation, apoptosis and autophagy of RA-FLSs. Additionally, we evaluated the effects of oridonin in combination with chloroquine (CQ), an inhibitor of autophagy, on RA-FLSs.

## Materials and methods

### Cell culture

RA-FLSs were obtained from patients admitted to the Shenzhen Nanshan People’s Hospital. The study was approved by the Institutional Research Ethics Committee of Shenzhen Nanshan People’s Hospital (approval no. 2017071950). Informed consent was provided by all participants prior to participation in the study. Tissue samples were cut into 4 × 4×2 mm fragments and maintained in a humidified chamber with 5% CO_2_ in low-glucose DMEM culture medium containing 10% FBS, 100 U/mL penicillin and 100 μg/mL streptomycin. For sub-culturing purposes, cells were detached using 0.05% trypsin–EDTA treatment at 37 °C.

### Cell treatments

For cell proliferation assay, cells were treated with various concentrations of oridonin (purity: 98% HPLC; O111381, Aladdin Bio-Chem Technology Co., Ltd., Shanghai, China) alone for the indicated times, or pre-treated with 100 µM CQ (C6628, Sigma, St. Louis, MO) for 30 min prior to oridonin treatment. For western blot analyses of ATG5 and Beclin1, cells were treated with 8 µg/mL oridonin for the indicated time. For western blot analyses of Bax and caspase-3, cells were treated with 8 µg/mL oridonin for 24 h. For annexin V-FITC apoptosis assay, cells were incubated with 8 µg/mL oridonin alone for the indicated time, or pre-treated with 100 µM CQ for 30 min before oridonin treatment. For enzyme-linked immunosorbent assay (ELISA), cells were treated with 8 µg/mL oridonin for 24 h.

### CCK8 assays

Cell proliferation was assessed using a CCK-8 Kit (Beyotime, Shanghai, China). Cells were seeded in 96-well plates at a density of 1 × 10^3^ cells/well 24 h prior to treatment. The treated cells were stained with 10% CCK-8 reagent at 37 °C for 4 h. Cell proliferation was measured by absorbance at a wavelength of 450 nm. Three biological replicates were evaluated.

### Western blot

The treated cells were washed with cold PBS, resuspended with RIPA buffer containing proteinase and phosphatase inhibitors (Sigma-Aldrich, St. Louis, MO), and lysed at 4 °C for 1 h. The protein concentration was determined using a Pierce™ BCA Protein Assay Kit (Thermo Fisher, Waltham, MA). Equivalent amounts of total protein were separated on 12% gels by SDS-PAGE electrophoresis and transferred to PVDF membranes. The membranes were blocked with 5% defatted milk for 1 h and then immunoblotted with primary antibody anti-ATG5 (#12994, Cell Signaling Technology, Danvers, MA), anti-Beclin1 (#37385, Cell Signaling Technology, Danvers, MA), anti-Bax (#2774, Cell Signaling Technology, Danvers, MA), anti-caspase-3 (#9662, Cell Signaling Technology, Danvers, MA) or anti-GAPDH (#1310016, Thermo Fisher Scientific, Waltham, MA) overnight at 4 °C. The membranes were washed and then incubated with specific secondary antibodies (#2999, Cell Signaling Technology, Danvers, MA). Finally, the blots were visualized by chemiluminescence (ECL; Forevergen Biosciences Center, Guangzhou, China).

### Annexin V-FITC apoptosis assay

Apoptosis was quantified using an FITC Annexin V Apoptosis Detection Kit (BD Pharmingen, San Diego, CA) according to the manufacturer’s instructions. A total of 5000 cells were analysed by flow cytometry, and the data were analysed using CellQuest software (BD Bioscience, San Diego, CA).

### ELISA

The IL-1β levels in the cell supernatants were determined using a human IL-1β ELISA Kit (Cusabio, Wuhan, China) as per the manufacturer’s protocol.

### Autophagy analysis

RA-FLSs were seeded in six-well plates and transfected with 2 µg GFP-LC3 plasmid using Lipofectamine 2000 reagent (Invitrogen, Waltham, MA) according to the manufacturer’s protocol. After 24 h, the cells were either left untreated or treated with 100 µM CQ for 30 min, followed by treatment with 8 µg/mL oridonin for an additional 24 h. The cells were observed under an inverted fluorescence microscope (ZEISS, Oberkochen, Germany). GFP-LC3 punctate dots per cell in GFP-positive cells were counted in five different visual areas per well. Autophagy was assessed based on the number of GFP-LC3 punctate dots per GFP-positive cell.

### Statistical analysis

All experiments were repeated at least three times. Data are presented as means ± SD. Student’s *t*-test (unpaired, two-tailed) analyses were performed to evaluate differences between groups. *p* Values of <0.05 were considered statistically significant.

## Results

### Oridonin inhibits proliferation of RA-FLSs

**Figure 1.**
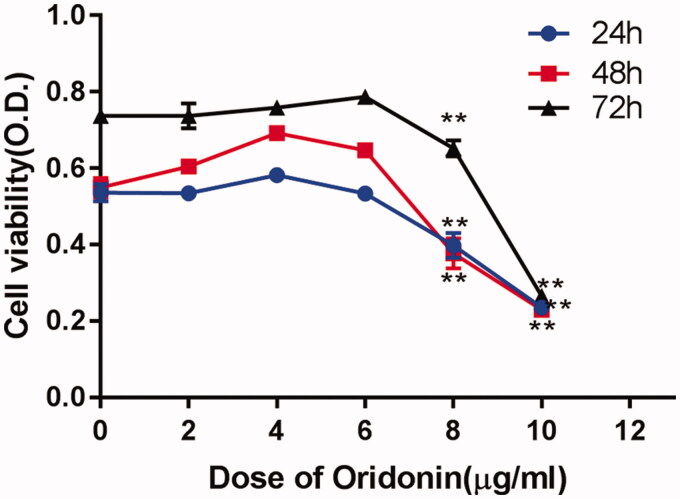
Effect of oridonin on proliferation of RA-FLSs. Proliferation of RA-FLSs was determined by CCK-8 assay. RA-FLSs were treated with oridonin at serial concentrations of 0, 2, 4, 6, 8 and 10 µg/mL for 24, 48 and 72 h, respectively. **p < 0.01.

To investigate the effect of oridonin on RA-FLS proliferation, a CCK-8 assay was performed. RA-FLSs were treated with oridonin at serial concentrations of 0, 2, 4, 6, 8 and 10 µg/mL for 24, 48 and 72 h. The assay results indicated that oridonin suppressed RA-FLS proliferation in a dose-dependent but not time-dependent manner. In addition, treatment with 8 µg/mL oridonin for 24, 48 and 72 h significantly decreased cell proliferation (Figure 1). The half maximal inhibitory concentrations (IC_50_) of oridonin at 24, 48 and 72 h were 8.28, 7.88 and 8.35 µg/mL, respectively. Therefore, 8 µg/mL oridonin was used in subsequent experiments.

### Oridonin suppresses autophagy in RA-FLSs

**Figure 2.**
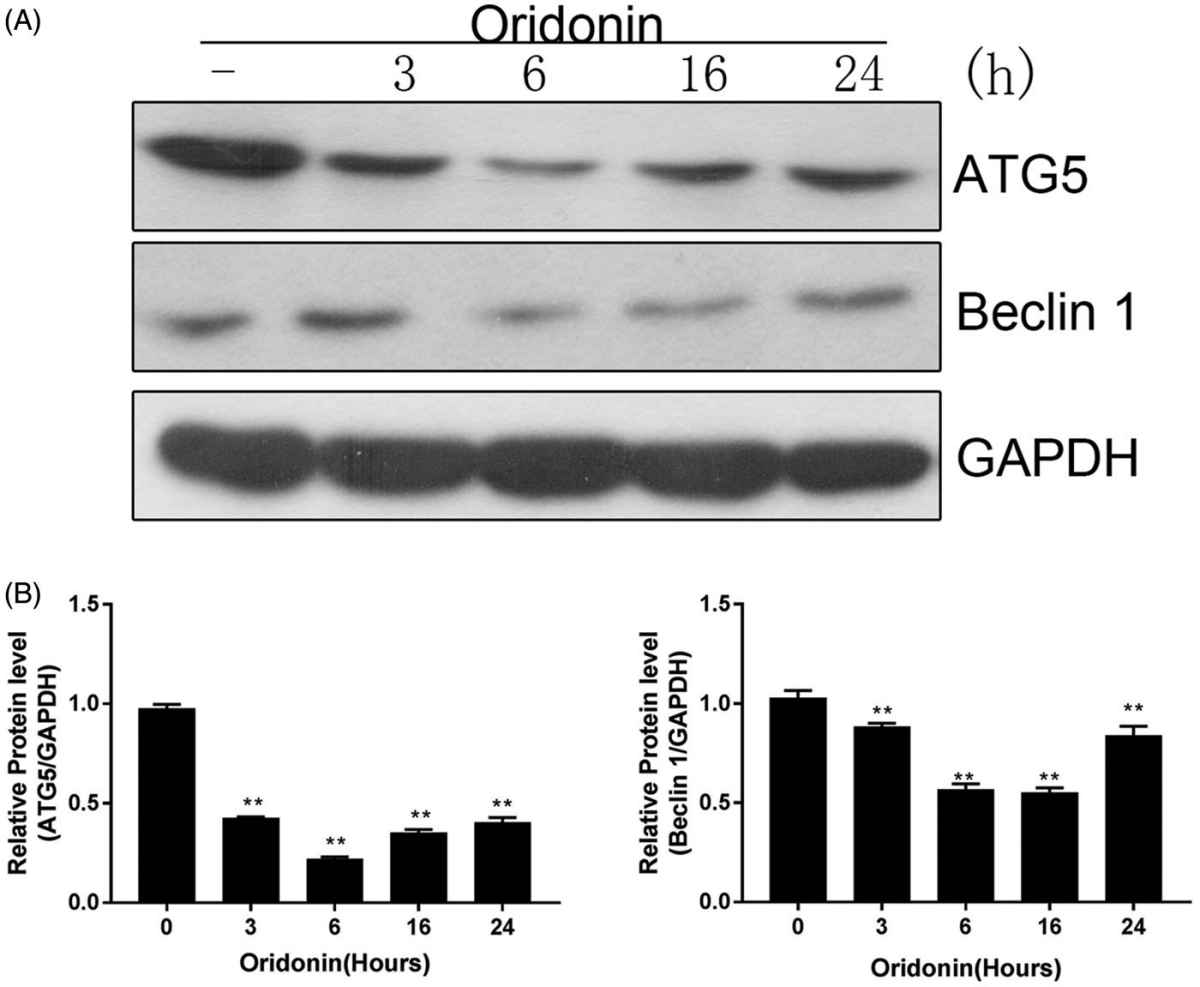
Effect of oridonin on autophagy in RA-FLSs. Cells were exposed to 8 µg/mL oridonin for 3, 6, 16 and 24 h. (A) ATG5 and Beclin1 protein levels were detected by western blot. (B) ATG5 and Beclin1 expression levels. GAPDH was used as an internal standard. **p < 0.01.

To assess the status of autophagy in oridonin-treated RA-FLSs, the cells were exposed to 8 µg/mL oridonin for 3, 6, 16 and 24 h. The protein levels of ATG5 and Beclin1 in treated cells were detected via western blot. Oridonin dramatically decreased the levels of ATG5 and Beclin1 in RA-FLSs (Figure 2(A)). Six hours post-treatment, the protein levels of ATG5 and Beclin1 were inhibited by 80.01% and 42.12%, respectively (Figure 2(B)). The decreased ATG5 and Beclin1 protein levels suggest that autophagy was disrupted in oridonin-treated RA-FLSs.

### Oridonin stimulates apoptosis and decreases in IL-1β levels RA-FLSs

**Figure 3.**
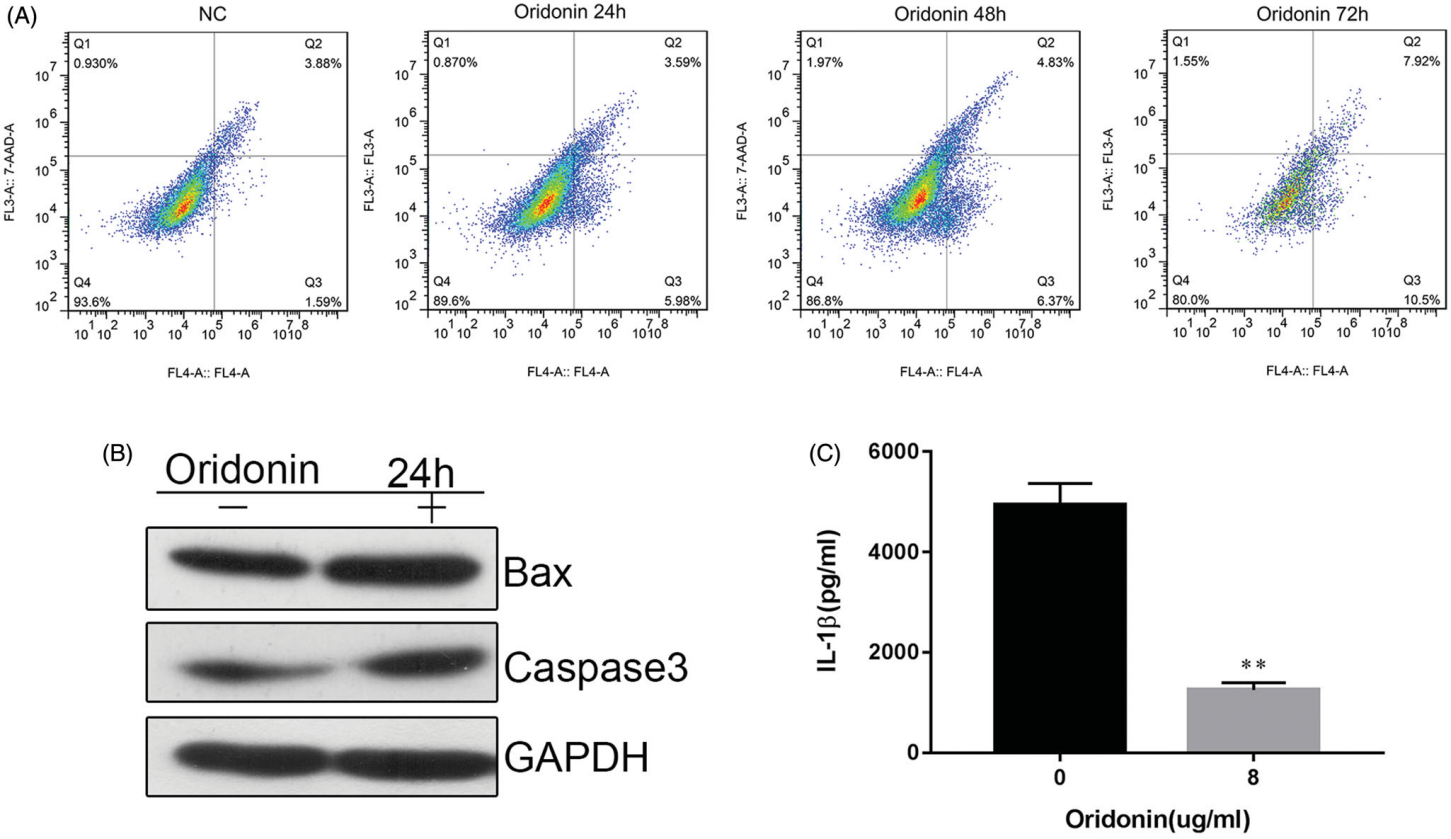
Effect of oridonin on apoptosis in RA-FLSs. Cells were treated with 8 µg/mL oridonin for 24, 48 and 72 h. (A) Apoptosis was assessed by flow cytometric analysis. (B) Bax and cleaved caspase-3 protein levels in untreated and oridonin-treated RA-FLSs were determined by western blot. (C) IL-1β level in the culture supernatant was detected by ELISA. **p < 0.01.

To explore whether oridonin stimulates apoptosis in RA-FLSs, the cells were treated with 8 µg/mL oridonin for 24, 48 and 72 h, and then flow cytometric analysis was performed to assess apoptosis. Oridonin increased apoptosis by 4.1% at 24 h post-treatment (Figure 3(A)). Increasing the treatment time had no effect on oridonin-induced apoptosis. Moreover, the protein levels of Bax and cleaved caspase-3 in oridonin-treated cells were elevated after 24 h of treatment (Figure 3(B)). In addition, oridonin caused a significant decrease in the production of the levels of the pro-inflammatory cytokine IL-1β in the culture supernatant (Figure 3(C)).

### Autophagy inhibitor (CQ) potentiates oridonin-induced apoptosis and suppression of proliferation in RA-FLSs

**Figure 4.**
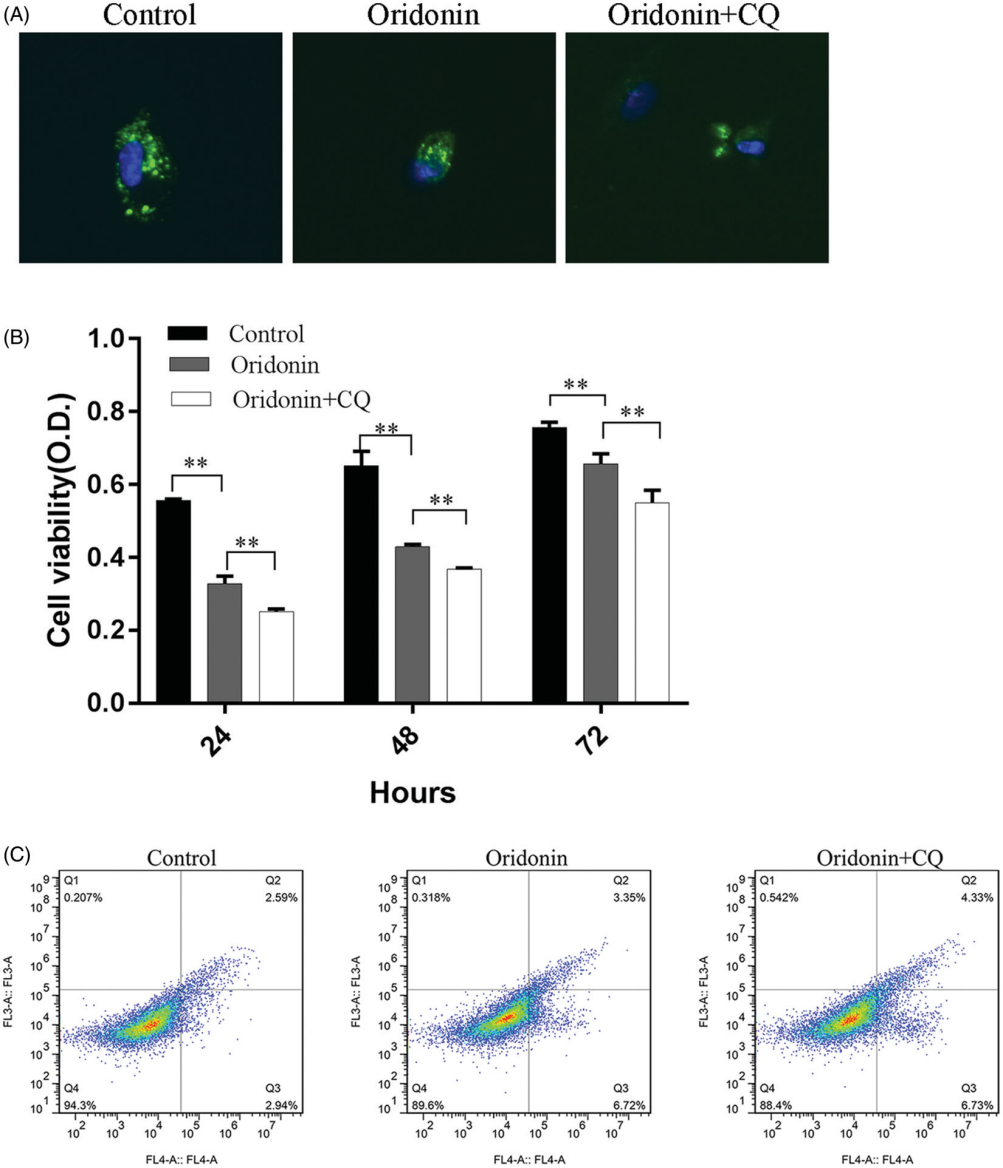
Effect of oridonin and CQ on RA-FLSs. Cells were treated with 100 µM CQ for 30 min and then with 8 µg/mL oridonin for 24 h. (A) A GFP-LC3 plasmid was transfected into the cells 24 h prior to combined oridonin and CQ treatment, and autophagy was measured by counting the number of GFP-LC3 punctate dots per GFP-positive cell. (B) A CCK-8 assay was performed to detect proliferation. (C) Flow cytometry was performed to detect apoptosis in treated and untreated cells. **p < 0.01.

To evaluate the effect of oridonin in combination with CQ on RA-FLSs, GFP-LC3 was transfected into RA-FLSs for 24 h, and then cells were treated with 100 µM CQ for 30 min, followed by treatment with 8 µg/mL oridonin for 24 h. Autophagy was measured by counting the number of GFP-LC3 punctate dots per cell. Oridonin treatment significantly decreased the number of GFP-LC3 punctate dots per cell compared to untreated cells, and pre-treatment with CQ combination enhanced this effect (Figure 4(A)). To determine whether CQ enhanced the suppression of proliferation and promoted the induction of apoptosis following oridonin treatment, CCK-8 and flow cytometry were performed to detect proliferation and apoptosis. CQ pre-treatment markedly potentiated the inhibitory effect of oridonin on RA-FLS proliferation (Figure 4(B)). The results of flow cytometry suggest that treatment with a combination of CQ and oridonin increased apoptosis from 10.07% (oridonin only treatment) to 11.06% (combined treatment) (Figure 4(C)). These results indicate that CQ enhanced the effects of oridonin on the suppression of proliferation and induction of apoptosis in RA-FLSs.

## Discussion

The intimal lining of the synovium, inside the fibrous outer layer, comprises predominantly two cell types: macrophage-like synoviocytes and FLSs (Mor et al. 2005). The role of FLSs in the onset and progression of RA is well established (Bartok and Firestein 2010). FLSs are implicated in several pathological aspects of RA via the promotion of synovitis and pannus growth, and ultimately cartilage/bone destruction (Bartok and Firestein 2010).

In patients with RA, FLSs undergo hyperplasia due to dysregulated proliferation and compromised autophagy. The capacity of RA-FLSs for hyper-survival is considered a tumour-like characteristic and leads to the formation of an aggressive pannus within the synovium (Firestein 1996; Bartok and Firestein 2010).

Oridonin stimulates apoptosis and suppresses proliferation in a variety of cancer cells, such as prostate, breast and non-small cell lung cancer cells (Ikezoe et al. 2003; Li et al. 2011). In the present study, we found that oridonin suppresses proliferation of RA-FLSs in a dose-dependent but not time-dependent manner.

Autophagy plays an important role in homeostasis, mediating the digestion and recycling of cytoplasmic organelles, proteins and macromolecules (Feng et al. 2018). The generation of autophagosomes is a hallmark of autophagy (Mizushima 2007). Beclin1 plays a critical role in the initial phase of autophagy (Parzych and Klionsky 2014); complexes involving PI3KCIII with either Beclin1-UVRAG or Beclin1-Ambral activate autophagy and initiate nucleation (Parzych and Klionsky 2014). During autophagophore elongation, the Atg12–Atg5–Atg16L complex acts as an E3-ubiquitin-like ligase, conjugating phosphatidylethanolamine to LC3-I and converting it to LC3-II. Then, LC3-II attaches to the surfaces of autophagophores and promotes autophagophore expansion (Hurley and Young 2017). In the synovial tissues of patients with RA, autophagy is enhanced, and the protein levels of Beclin1 and LC3 are upregulated (Xu et al. 2013; Zhu et al. 2017). To explore the effects of oridonin on autophagy in RA-FLSs, we determined the protein levels of ATG5 and Beclin1 in treated and untreated cells. Our results show that oridonin treatment dramatically downregulated ATG5 and Beclin1 levels and impaired autophagy in RA-FLSs.

Apoptosis is the mechanism by which cells undergo genetically programmed regulated self-elimination that functions as a homeostatic mechanism to maintain cell populations in tissues and is a defence mechanism against damaged cells (Elmore 2007). Several studies indicate a connection between dysregulated apoptosis and RA (Xu et al. 2013; Jiao et al. 2018; Kim et al. 2018). Resistance to apoptosis has been observed in RA-FLSs. Therefore, we assessed levels of apoptosis in oridonin-treated cells. Our results demonstrate that oridonin enhanced apoptosis. Consistent with the proliferation results, the induction of apoptosis was dose-dependent but not time-dependent; there was no significant increase in apoptosis in cells treated for 48 or 72 h compared with those treated for 24 h.

The crosstalk between apoptosis and autophagy is complicated and experimental results on the topic are often contradictory. This crosstalk can be categorized into three main types: cooperative apoptosis and autophagy to induce cell death; autophagy that blocks apoptosis by promoting cell survival; and autophagy that enables apoptosis to induce cell death without autophagocytic cell death (Booth et al. 2014; Marino et al. 2014). In RA-FLSs, multiple mechanisms may contribute to compromised apoptosis, including increased anti-apoptotic factors (Kurowska et al. 2002; Jiao et al. 2018), suppressed pro-apoptotic factors (Garcia et al. 2010) and increased autophagy (Dai and Hu 2016). Reduced apoptosis correlates with enhanced autophagy in the synovial tissues of patients with RA (Xu et al. 2013). Further, it has been suggested that in fibroblasts from patients with RA, autophagy protects cells from death by alleviating the ER stress response (Xu et al. 2013). Therefore, we hypothesized that CQ could enhance the effects of oridonin on the suppression of RA-FLS survival. Our results show that treatment with a combination of CQ and oridonin inhibits autophagy and cell proliferation and induces apoptosis more effectively than oridonin alone.

## Conclusions

Oridonin can impair autophagy and proliferation, promote apoptosis, and attenuate IL-1β secretion in RA-FLSs. Autophagy inhibition with CQ significantly enhances the anti-survival effects of oridonin. The findings of the present study indicate that oridonin is a potential therapeutic agent for RA, although the topic requires further research.
